# The diversity of decay

**DOI:** 10.7554/eLife.60375

**Published:** 2020-08-04

**Authors:** Emma J Sayer, Ralf Schäfer

**Affiliations:** 1 Lancaster Environment Centre, Lancaster UniversityLancasterUnited Kingdom; 2 Institute for Environmental Sciences, University Koblenz-LandauLandauGermany

**Keywords:** biodiversity, ecosystem functioning, meta-analysis, litter decomposition, plant species richness, carbon and nutrient cycles, Other, None

## Abstract

To predict how species loss will affect ecosystems, it is important to consider how biodiversity influences processes such as decomposition.

**Related research article** Beaumelle L, De Laender F, Eisenhauer N. 2020. Biodiversity mediates the effects of stressors but not nutrients on litter decomposition. *eLife* **9**:e55659. doi: 10.7554/eLife.55659

**Related research article** Kou L, Jiang L, Hättenschwiler S, Zhang M, Niu S, Fu X, Dai X, Yan H, Li S, Wang H. 2020. Diversity-decomposition relationships in forests worldwide. *eLife* **9**:e55813. doi: 10.7554/eLife.55813

Decaying leaves are nature’s compost: they help to form organic matter in the soil, and they provide nutrients to plants and decomposers (such as invertebrates, bacteria and fungi) on land and in water (Webster, 2007). Biodiversity plays an important role in these processes. The leaves of different plant species have distinct chemical and physical properties, and thus cater to diverse communities of invertebrate and microbial decomposers (Swift et al., 1979; Graça, 2001). Since the decay of plant litter is controlled by complex interactions between plants, invertebrates and microbes, biodiversity loss as a result of human activity could strongly affect decomposition (Gessner et al., 2010).

In theory, plant diversity and decomposer diversity are linked because mixed plant litter provides a wider range of resources to many different groups of organisms. In practice, however, experiments show variable effects – litter diversity can either promote or impede decomposition, or have no effect at all (Kampichler and Bruckner, 2009; Hättenschwiler et al., 2005; Gessner et al., 2010; Tank et al., 2010). Human activities are altering ecosystems at an alarming rate and the consequent loss of species is rarely random, but instead affects some species or groups more than others (Duffy, 2003). To assess how a reduction in biodiversity affects the ecosystem, more information on the habitats and species that are most vulnerable to change is needed.

Now, in eLife, two independent meta-analyses report how biodiversity influences decomposition processes in different contexts. Liang Kou and colleagues – including Lei Jiang as joint first author and Shenggong Li and Huimin Wang as joint corresponding authors – analyzed the relationship between the diversity of plant litter and decomposition across 65 field studies in forests around the world (Kou et al., 2020). Léa Beaumelle, Frederik De Laender and Nico Eisenhauer studied the effect of fertilizers and toxic chemicals on decomposer diversity and decomposition across 69 studies (Beaumelle et al., 2020). Both meta-analyses highlight important links between litter diversity and decomposer diversity, with Beaumelle et al. – who are based at institutes in Germany and Belgium – also demonstrating that these links can be influenced by human activity.

Kou et al. – who are based in China and France – found that the influence of plant litter diversity was strongest during the first few months of decomposition, possibly because a wider variety of resources was available to the decomposers (Whalen and Sampedro, 2010). Nutrients, such as nitrate and phosphate, are a valuable resource to decomposers, and Beaumelle et al. found that adding these minerals increased decay, even when decomposer diversity was low. The importance of nutrients is also apparent in the finding by Kou et al. that faster decomposition of diverse litters was related to higher variation of certain leaf nutrients that are particularly important to some decomposer species. The availability of nutrients probably influences decay by promoting competition among different groups of organisms, allowing some decomposers to multiply at the expense of others. However, there can be too much of a good thing: high levels of nutrients were sometimes associated with reduced diversity and slower decomposition rates, possibly because some nutrient sources can also include toxic chemicals (Beaumelle et al., 2020; Woodward et al., 2012).

Both studies demonstrate that the influence of biodiversity on decomposition can vary with habitats, with groups of organisms, and with various stressors associated with human activities. For example, by analyzing decomposition in forests worldwide, Kou et al. established that the effects of increased litter diversity were much weaker in the tropics and subtropics than in temperate and boreal forests. The strong influence of diversity on decomposition at high latitudes suggests that temperate and boreal forests may be more vulnerable to species loss than more diverse ecosystems such as tropical forests. Consequently, the impact of human-induced loss of biodiversity on decomposition processes will also vary widely across the globe. Moreover, Beaumelle et al. revealed that the diversity of animal decomposers was much more strongly affected by both toxic chemicals and elevated nutrients than microbial diversity, indicating that human activities can affect some organisms more than others. Many other ecosystem functions also involve interactions among several groups of organisms. It is thus difficult to predict the consequences of species losses as a result of human activities.

In conclusion, the studies by Kou et al. and Beaumelle et al. demonstrate that decomposition processes are influenced by complex interactions among different groups of organisms. The type and magnitude of human activities can alter the biodiversity of each of these groups, which in turn influences decomposition (Figure 1). To understand how human-induced biodiversity loss will affect important ecosystem processes, we need to integrate research across many individual components of ecosystems, including plants, animals and microbial communities, and do so in a way that allows to compare change across different ecosystems.

**Figure 1.**
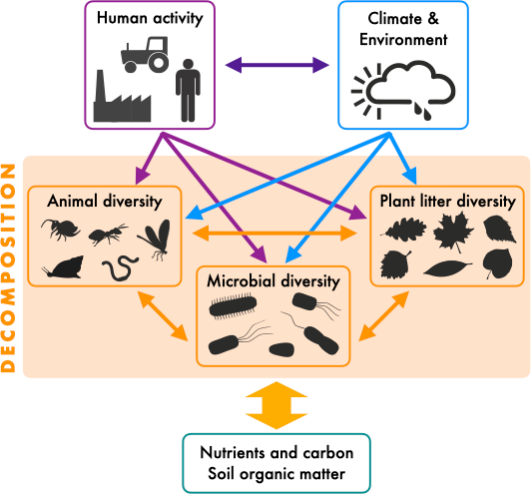
The effects of biodiversity on litter decomposition. The diagram illustrates how the decay of plant litter is driven by diversity, which in turn is influenced by human activities (purple) and environmental factors (blue). Changes in the diversity of plant litter, animal decomposers or microbial decomposers can alter these processes (orange), ultimately affecting ecosystem carbon and nutrient cycles. Single arrows indicate influences and double arrows indicate interactions or feedbacks.

## References

[bib1] Beaumelle L, De Laender F, Eisenhauer N (2020). Biodiversity mediates the effects of stressors but not nutrients on litter decomposition. eLife.

[bib2] Duffy JE (2003). Biodiversity loss, trophic skew and ecosystem functioning. Ecology Letters.

[bib3] Gessner MO, Swan CM, Dang CK, McKie BG, Bardgett RD, Wall DH, Hättenschwiler S (2010). Diversity meets decomposition. Trends in Ecology & Evolution.

[bib4] Graça MA (2001). The role of invertebrates on leaf litter decomposition in streams–a review. International Review of Hydrobiology.

[bib5] Hättenschwiler S, Tiunov AV, Scheu S (2005). Biodiversity and litter decomposition in terrestrial ecosystems. Annual Review of Ecology, Evolution, and Systematics.

[bib6] Kampichler C, Bruckner A (2009). The role of microarthropods in terrestrial decomposition: a meta-analysis of 40 years of litterbag studies. Biological Reviews.

[bib7] Kou L, Jiang L, Hättenschwiler S, Zhang M, Niu S, Fu X, Dai X, Yan H, Li S, Wang H (2020). Diversity-decomposition relationships in forests worldwide. eLife.

[bib8] Swift MJ, Heal OW, Anderson JM, Anderson JM (1979). Decomposition in Terrestrial Ecosystems.

[bib9] Tank JL, Rosi-Marshall EJ, Griffiths NA, Entrekin SA, Stephen ML (2010). A review of allochthonous organic matter dynamics and metabolism in streams. Journal of the North American Benthological Society.

[bib10] Webster JR (2007). Spiraling down the river continuum: stream ecology and the U-shaped curve. Journal of the North American Benthological Society.

[bib11] Whalen JK, Sampedro L (2010). Soil Ecology and Management.

[bib12] Woodward G, Gessner MO, Giller PS, Gulis V, Hladyz S, Lecerf A, Malmqvist B, McKie BG, Tiegs SD, Cariss H, Dobson M, Elosegi A, Ferreira V, Graça MA, Fleituch T, Lacoursière JO, Nistorescu M, Pozo J, Risnoveanu G, Schindler M, Vadineanu A, Vought LB, Chauvet E (2012). Continental-scale effects of nutrient pollution on stream ecosystem functioning. Science.

